# Reconciling cooperation, biodiversity and stability in complex ecological communities

**DOI:** 10.1038/s41598-019-41614-2

**Published:** 2019-04-03

**Authors:** Chengyi Tu, Samir Suweis, Jacopo Grilli, Marco Formentin, Amos Maritan

**Affiliations:** 1 School of Ecology and Environmental Science, Yunnan University, 650091 Kunming, China; 2 Department of Environmental Science, Policy, and Management, University of California, Berkeley, CA 94720-3114 USA; 3 Department of Physics and Astronomy “Galielo Galilei”, University of Padova, Via Marzolo 8, 35131 Padova, Italy; 4 Santa Fe Institute, 1399 Hyde Park Road, Santa Fe, NM 87501 USA; 5 The Abdus Salam International Centre for Theoretical Physics (ICTP), Strada Costiera 11, 34014 Trieste, Italy; 6 Department of Mathematics “Tullio Levi-Civita”, University of Padova, Via Trieste 63, 35129 Padova, Italy

## Abstract

Empirical evidences show that ecosystems with high biodiversity can persist in time even in the presence of few types of resources and are more stable than low biodiverse communities. This evidence is contrasted by the conventional mathematical modeling, which predicts that the presence of many species and/or cooperative interactions are detrimental for ecological stability and persistence. Here we propose a modelling framework for population dynamics, which also include indirect cooperative interactions mediated by other species (e.g. habitat modification). We show that in the large system size limit, any number of species can coexist and stability increases as the number of species grows, if mediated cooperation is present, even in presence of exploitative or harmful interactions (e.g. antibiotics). Our theoretical approach thus shows that appropriate models of mediated cooperation naturally lead to a solution of the long-standing question about complexity-stability paradox and on how highly biodiverse communities can coexist.

## Introduction

Research in population dynamics has a long history dating back to almost one thousand years ago with Fibonacci’s modeling of the rabbit population. Nevertheless, it is still under debate which are the mechanisms allowing the coexistence of many interacting species in the same environment^1–3^. The current loss of Earth’s biodiversity^4^ makes this open question of great relevance today more than ever, and this challenge calls for interdisciplinary approaches. Historically, the Lotka and Volterra (LV) equations have provided much theoretical guidance and several microscopic derivations of these equations have been proposed^1,5–7^. Furthermore, these equations are the core of most of the multi-species deterministic population dynamics models based on the ecological concept of niche partitioning: competing species in order to coexist need to interact with the environment differently and to rely on not-overlapping resources. Recent works^8–10^ have studied the conditions for persistence of many species in random Lotka-Volterra equations, obtaining a trade-off between diversity and strength of interactions.

While consumer-resource dynamics and related models of population dynamics with prey-predator and competitive interactions have been extensively studied, cooperative interactions (mutualism and/or commensalism), which are beneficial to one or both the involved species, have historically received less attention^11^. The current approach to mutualistic population dynamics is a simple extension of the LV types of models, which does not change the functional form of the two-species interaction in the phenomenological equations. Indeed beneficial (+ +) instead of predator-prey (+ −, here called exploitative) interactions are utilized^1,12^. In particular, a microscopic derivation of the phenomenological equations specific for the population dynamics in mutualistic communities is still missing. Moreover, a generalization of the stability-complexity theorem^1,13^ has revealed that mutualism is even more detrimental to stability as the product *S* × *c* increases^3,14–16^, where *S* is the number of species and *c* the connectivity, i.e. the fraction of non-zero pairwise interactions between species. This prediction clashes with the observation of widespread mutualistic interactions (or other cooperative interactions) in many natural and laboratory communities and where the biodiversity is very high^17–20^, although other cases have been also reported^21^.

**Figure 1:**
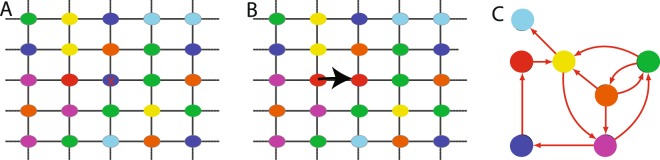
Graphical representation of our model. (**A**,**B**) The population dynamics is stochastic: at each time step a randomly chosen individual of a given species (denoted by the color) die and it is replaced by another species that is also picked at random and give birth to an offspring. The species birth rate depends on the species population abundance and on the species interaction matrix (**C**) - as given by Eq. (1).

An alternative theoretical approach to niche-based multi-species deterministic modeling is the Neutral Theory (NT) of Biodiversity^2,22,23^. In NT organisms of a community have identical per-capita probabilities of giving birth, dying, migrating, and speciating, regardless of the species they belong to. In this sense, NT is symmetric and aims to model only species on the same trophic level, therefore competing for the same pool of resources. An important example of the neutral model is the voter model (VM)^24^. VM was initially introduced to model the dynamics of two or more competing opinions of a group of people and its evolution in a discrete time setting can be described as follow: at each time step a randomly chosen individual changes his/her opinion to agree with one of her/his neighbors picked at random (see Fig. 1). In the ecological context one deals with a community of *N* individuals belonging to *S* different species. In its simplest version, a randomly selected individual dies and the corresponding resources are freed up for colonization by other species at the rate proportional to their abundances. An important limitation of this modeling is that, apart from competition, it does not explicitly consider species interactions (e.g. mutualism/commensalism). Although it has been already shown that niche and neutral approaches are only apparently contrasting^25,26^, and few stochastic models have studied the impact of cooperative interactions on species coexistence and biodiversity patterns^19,27^, two crucial issues in the current literature are: (i) the lack of a general framework specifically developed to model indirect (e.g. habitat modification or cross-feeding) cooperative interactions; (ii) the role of cooperative interactions in determining species coexistence and how they impact on patterns such as ecosystem stability^3,14,28^.

In this work, we thus present a theoretical framework where, starting from a VM-like microscopic stochastic modelling, we add interactions among species and we properly account for the effect of the different type of ecological interactions. These interactions affect neutrality and lead, in their mean field formulation, to an emergent multi-species (implicit resource) model where cooperation is specifically modelled to take in account niche construction (by habitat modification through, for example, of release of secreted compounds). Reconciling apparently contrasting observations and previous results^1,3,13–15^, we show that, in our model ecosystem, cooperation promotes biodiversity and diversity increases stability.

## Results

### Voter model with ecological interactions: breaking neutrality

In details, let be *η*_*z*_ the species label at spatial position *z*, where *η*_*z*_ ∈ {1, …, *S*} and *z* = 1, …, *N*. The state at time *t* of the system is given by *η*(*t*) = (*η*_1_(*t*), *η*_2_(*t*), …, *η*_*N*_(*t*)) ∈ {1, …, *S*}^*N*^. We also set ${\bar{\eta }}^{k}$ to be the fraction of individuals of the *k*-species present in the system. Both intra- and inter-species competition is indirectly accounted by fixing an hard constraint on the system carrying capacity^2,23^, i.e. an organism comes into the system always at the expenses of another organism. We now consider two main types of explicit ecological relationships: predator-prey or exploitative interactions (+ −) and cooperation (+ +, + 0). In the following we will also study the effect of harmful relationships (− 0) that can be mediated by secreted compounds (e.g. fosfomycin in bacteria communities).

We thus introduce a directed graph on the set {1, …, *S*}, where the nodes correspond to species and directed links represent the network of species interactions. Such a network is defined through cooperation matrix *M*_*ij*_ and exploitation matrix *L*_*ij*_ satisfying the following conditions: (i) For all *i*, *j* = 1, …, *S*, *M*_*ij*_ ≥ 0; (ii) For all *i*, *j* = 1, …, *S*, it must be *L*_*ij*_*L*_*ji*_ < 0 or *L*_*ij*_ = *L*_*ji*_ = 0; (iii) For all *i*, *j* = 1, …, *S*, we have *L*_*ij*_*M*_*ij*_ = 0, i.e. species *i* and *j* cannot simultaneously have both mutualistic and exploitative interactions.

In ecological terms, given two species *i* and *j*, a directed link of strength *M*_*ij*_ from *i* to *j* means that the *j*-th species receives a beneficial effect from the interaction with the *i*-th species, while *L*_*kl*_ > 0(<0) and *L*_*lk*_ < 0(>0) denotes that the *l*-th species exploits (is exploited by) the *k*-th species. For instance, in the former case we can think a microbial community where the presence of a certain species creates an environment by secreting metabolites, which modifies the niches and favors the growth of other bacteria^20^; in the latter one, we may think to host-parasite symbiosis. Typically, it is very difficult to measure the strength of the interactions among two species, so we adopt the standard approach of drawing the matrix entries from a given bivariate probability distribution (e.g. Gaussian or Uniform) in the same spirit as traditionally done^1,14^. If *Q* is a given matrix, we define *c*_*Q*_ its connectivity as well as *μ*_*Q*_, *σ*_*Q*_ and *ρ*_*Q*_ the mean, standard deviation and correlation of the *ij* and *ji* entries, respectively.

The dynamics is described by a continuum time stochastic Markov process: a randomly chosen individual is removed and substituted by an individual of the *j*-th species at a rate

1 $$\omega (j,\eta ,M,L)={\bar{\eta }}^{j}+{\epsilon }_{1}\sum _{k=1}^{S}{\bar{\eta }}^{k}{M}_{kj}\theta ({\bar{\eta }}^{j})+{\epsilon }_{2}\sum _{k=1}^{S}{\bar{\eta }}^{k}{L}_{kj}{\bar{\eta }}^{j}$$

where ${\epsilon }_{1}$ > 0 and ${\epsilon }_{2}$ > 0 give the cooperation and exploitation intensity, and *θ*(·) is the Heaviside step function, i.e. *θ*(*x*) > 0 when *x* > 0 and 0 otherwise. The presence of the *θ*-function in the mutualistic contribution, guarantees that the transition rate is zero if the *j*-th species is extinct. For ${\epsilon }_{1}$ = ${\epsilon }_{2}$ = 0 we recover the standard VM. When ${\epsilon }_{1}$ > 0 the species *j* is favored by the presence of the other species (*k* in the summation) to which it is connected and by their population. On the other hand, ${\epsilon }_{2}$ > 0 allows the possibility that a species exploits (or is exploited by) one or more other species.

It is important to highlight the differences of the contribution on Eq. (1) between exploitative and cooperative interactions. In the first case, the interaction term is quadratic in $\bar{\eta }$ (i.e. ${\bar{\eta }}^{k}{L}_{kj}{\bar{\eta }}^{j}$), as exploitative interactions can be derived using the law of mass-action used to describe chemical reactions^5,6^: a contact must occur between species and the chance of this interaction is, in the simplest hypothesis, proportional to both species concentrations. On the other hand, in cooperative interactions used to model effect of habitat modification or resource exchanges have a linear contribution to the birth rate in $\bar{\eta }$ (i.e. ${\bar{\eta }}^{k}{M}_{kj}$). In fact, if some species can generate a favorable environment for the growth of other species (like for example anaerobic conditions by aerobes, or the widespread phenomenon of growth facilitation through released metabolites driving non-limiting species co-occurrence^20^), then the linear term present in our equations just takes into account that the growth promoting effects are proportional to the population density of the species creating these conditions, but not to the population density of the species profiting of them. A further mechanism possibly behind the linear interaction term is indeed when interspecific cross-feeding (e.g. pollen, faecal pellets, metabolic waste) is present. Indeed, if a species is favored by a secreted compound released by another species, then what really contributes to the birth rate of the former is the amount of proper resources in the environment, which is proportional to the abundance of the latter (we assume that these resources are always fully utilized in the community and do not consider the limiting case of very low densities). If a species generates nourishment for more than one species, say *k* species, the hypothesis under which this leads to the above mentioned linear interaction term is when the secreted compound is used by each of the *k* species at a different time (see Supplementary Information, Section S1). This hypothesis has been used in many studies in cases where microbes tend to utilize nutrients in a specific sequential order^29^, as observed in Bacteroide species^30^ and in other studies^29^. Additionally, we also provide a mathematical derivation of the linear contribution to the birth rate based on the above mechanisms (see Supplementary Information, Section S1).

The microscopic dynamics given by rates Eq. (1) induces a Markovian evolution on the relative abundance ${\bar{\eta }}^{s}$ of each species. Standard techniques^31^ can be used to prove that as *N* → ∞, the process ${({\bar{\eta }}^{1}(t),\ldots ,{\bar{\eta }}^{S}(t))}_{t\ge 0}$ weakly converges to the solution of the system of ordinary differential (mean field) equation:

2 $$\begin{array}{c}\frac{{\rm{d}}}{{\rm{d}}t}{\bar{\eta }}^{s}(t)={\epsilon }_{1}\,\sum _{k=1}^{S}{\bar{\eta }}^{k}(t){M}_{ks}\,\theta ({\bar{\eta }}^{s}(t))+{\epsilon }_{2}\,\sum _{k=1}^{S}{\bar{\eta }}^{k}(t){L}_{ks}{\bar{\eta }}^{s}(t)\\ \,\,\,\,-\,{\bar{\eta }}^{s}(t)({\epsilon }_{1}\,{\bar{\eta }}^{k}(t){M}_{ki}\,\theta ({\bar{\eta }}^{i}(t))+{\epsilon }_{2}\,{\bar{\eta }}^{k}(t){L}_{ki}\,{\bar{\eta }}^{i}(t))\end{array}$$

for *s* = 1, …, *S* where $\sum _{j=1}^{S}{\bar{\eta }}^{j}(t)=1$ and is conserved by the dynamics.

### Emergent ecological patterns

Through Eq. (2) we can study many ecosystem properties of interest. One of the most important and studied emergent pattern in ecology, which we can determine within our model, is the relative species abundance (RSA)^2,22,23^. It describes commonness and rarity of species, thus characterizing the biodiversity of an ecological community. In our model, the RSA is given by the mean field stationary solution (*m*_1_, …, *m*_*S*_), which in turn depends on the species interaction matrices *M* and *L*.

**Figure 2:**
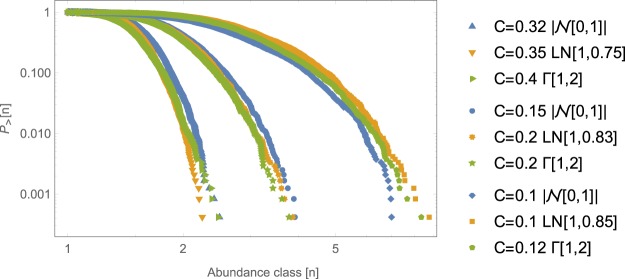
Cumulative RSA for a network of 300 species. The matrix elements of both ${M}_{ij}$ and ${L}_{ij}$ have been drawn from three different probability distributions (${z}_{h} \sim {p}_{h}(z)$, ${M}_{ij} \sim {z}_{h}$, ${L}_{ij} \sim {z}_{h}$, ${L}_{ji} \sim -{z}_{h}$, $h=1,2,3$): the modulus of a Normal distribution ${z}_{1} \sim |{\mathscr{N}}(\alpha ,\beta )|$ (blue lines), Gamma distribution ${z}_{2} \sim {\rm{\Gamma }}(\alpha ,\beta )$ (green lines) and LogNormal distribution ${z}_{3} \sim LN(\alpha ,\beta )$ (orange lines). Connectivity for mutualistic interaction, $M$, is denoted by ${C}_{M}=C$, while for exploitative interactions, $L$, is denoted by ${C}_{L}=0.1C$ (in all the studied cases ${C}_{M}+{C}_{L}\ge \,1$). The cooperative and exploitative intensities are ${\epsilon }_{1}={\epsilon }_{2}=1$. We set the distribution parameters $\alpha ,\beta$ (see legend) so that in each case we build interaction matrices with three different values of coefficient of variation ${\rm{CV}}\approx 2,3,4$. Log-log plots display the relative species abundances (RSA) from the stationary solution of Eq. (1) averages over 100 realizations and normalized so that the smallest population is 1. As we can see, the cumulative RSA is not very sensible to the distribution from which the matrix elements of both ${M}_{ij}$ and ${L}_{ij}$ are drawn, but only on the CV.

The cumulative RSA is thus defined as the fraction of species with population greater that a certain value *n*, ${P}_{ > }[n]=\frac{1}{S}\sum _{k=1}^{S}\theta (n-N{m}_{k})$ where we have fixed *N* = 1/min (*m*_1_, …, *m*_*S*_) when all species coexist, i.e. we have made the choice that the rarest species has population equal to 1. We numerically find that the stationary RSA displays a log-normal shape, as the one found in many real ecosystems^2^, and weakly depends on the specific distribution of the matrix elements *M*_*ij*_ and *L*_*ij*_ (see Fig. 2). Indeed, it is mainly determined only on its coefficient of variation, CV, i.e. the standard deviations of interaction strengths *M* and *L* relative to its mean (see Supplementary Information, Section S2). This allows to constrain the model parameters: in order to parametrize species interactions strengths, that are typically unknown, we use the random matrix approach^1,14,15^, where we fix the mean and the standard deviation according to the desired RSA one needs to fit. Our deterministic approximation for the RSA neglects the fluctuations due to demographic stochasticity, which on the other hand decreases as $1/\sqrt{N}$ and thus when *N* is large enough, its effect becomes negligible.

We now consider species abundance fluctuations, defined as ${x}_{N}^{i}(t)=\sqrt{N}({\bar{\eta }}^{i}(t)-{m}_{i})$ for *i* = 1, …, *S*. Another relevant quantity characterizing the ecosystem biodiversity is the covariance matrix *V*, ${V}_{ij}=\langle {x}^{i}(t){x}^{j}(t)\rangle -$ $\langle {x}^{i}(t)\rangle \langle {x}^{j}(t)\rangle$, describing the correlations in the population abundance fluctuations between pairs of species population abundances^32^. In our setting, we can compute analytically this quantity in the limit of normal fluctuations. The stochastic process $({x}_{N}^{1}(t),\ldots ,{x}_{N}^{S}(t))$ converges in distribution to a Gaussian Markov process *X* : = (*X*^1^(*t*), …, *X*^*S*^(*t*)), which solves the stochastic differential equation *dX* = *AX dt* + Φ*dB*_*t*_, where *B*_*t*_ is a *S*-dimensional Brownian motion, which corresponds to a *S*-dimensional Ornstein-Uhlenbeck process^31,33^. The analytical expressions for the matrices *A* and Φ in terms of the interaction matrices *M* and *L*, and of the equilibria, (*m*_1_, …, *m*_*S*_), of Eq. (2), are given in the Supplementary Information, Section S2. The covariance matrix, *V*, can be obtained by solving the following Lyapunov matrix equation *AV* + *V A*^*T*^ + ΦΦ^*T*^ = 0.

This quantity is typically measured from species population time series, through the Pearson (or other type of) correlations^34^. Moreover, in many studies once the threshold is set opportunely, it is used as an empirical proxy of the species interactions matrix^34,35^. In other words, many works assume that *M* + *L* can be approximated through *V*. Other works, applying maximum entropy approach, use *V*^−1^ as the quantity to describe the species interactions network^32^. However, we find that both *V* and *V*^−1^ are not good proxies of the species interactions matrix *M* + *L* (see Supplementary Information, Section S2). This result highlights the importance to properly infer interaction networks from data^34^ by considering a suitable model, which explicitly takes into account species interactions.

Our shift in the assumptions behind cooperative interactions enlightens some theoretical problematic aspect in order to explain species coexistence and ecosystem stability as observed in many real complex ecological communities. In fact, we now show that cooperation promotes species biodiversity and strongly stabilizes the ecosystem dynamics.

### The importance of mediated cooperation for preserving biodiversity

If we focus on the purely cooperative voter model (${\epsilon }_{1}$ = $\epsilon$ and ${\epsilon }_{2}$ = 0), we are able to analytically relate key dynamical features of Eq. (2) to the topology of the interaction matrix *M* and prove various results of ecological importance.

First, we show that the presence of non-supported species–the *i*-th species is non-supported if $\sum _{j}{M}_{ji}=0$ – inhibits coexistence equilibria of the whole ecological community. More precisely, if species *i* is non-supported by other species then at stationarity Eq. (2) implies that *m*_*i*_ = 0. The extinction of the *i*-th may create new unsupported species that goes to zero in the large time limit. Such a cascade of extinctions may eventually end only when $\sum _{j}{M}_{ji} > 0$ for all nodes/species *i* of the network (see Supplementary Information, Section S3). The elimination of nodes of the interaction network corresponding to all non-supported species will be called pruning in the following.

Furthermore, we have found sufficient conditions on the topology of the cooperative interaction matrix *M* for the existence of stable stationary states of Eq. (2). In fact, if *M* is irreducible, i.e. if for any node *i* we can reach any other node *j* through a path of oriented links (*k*, *l*) such that *M*_*kl*_ > 0, then the Perron-Frobenius (PF) theorem holds^36^ and there is a unique non-trivial stationary state (*m*_1_, …, *m*_*S*_) with only positive entries. This solution is proportional to the left eigenvector, *v*, of *M* corresponding to the eigenvalue of *M* with the largest modulus, which turns out to be non-degenerate, real and positive^36^, denoted by *α* in the following (and that for brevity we will refer to it as PF eigenvalue). In other words, if *M* satisfies the PF theorem, then *α* tells us how the stationary species abundances *m* are distributed (see Methods). The corresponding right eigenvector will be denoted by *w*, and it gives information on how press perturbations spread throughout the network^15^. All components of both *v* and *w* are strictly positive and ${m}_{i}={v}_{i}/\sum _{k}{v}_{k}$. An example of irreducible matrix *M* occurs when *M*_*ij*_ > 0 implies *M*_*ji*_ > 0 and the network has a single connected component. Many network architectures that have been observed in natural ecological communities satisfy this condition (e.g. hierarchical modular structure in mutualistic networks)^18^.

**Figure 3:**
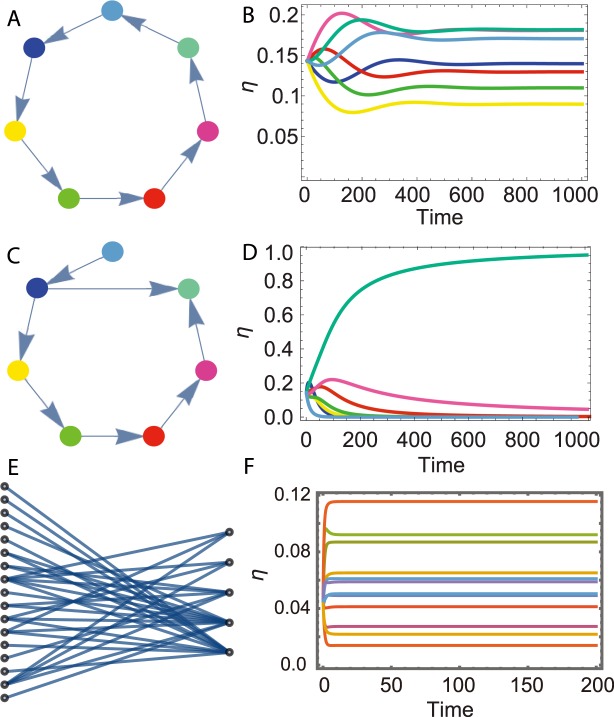
The results of the mean field predictions. (**A**) Species interaction network for 7 species where each species *i* has one mutualistic partner *j*, i.e. *M*_*ij*_ = 1, ${\epsilon }_{1}$ = 1, ${\epsilon }_{2}$ = 1. (**B**) Time evolution of the populations of the 7 species as predicted by the mean field dynamics Eq. (2). (**C**) Species interaction network for 7 species where one species is not helped by any species and the iterative pruning process, as described in the main text, leads to a cascade of extinctions. (**D**) As the time evolution of the mean field Eq. (2), only one species dominates the community. (**E**) Nested structure for fruit eating birds community in Mexico^39^. (**F**) All species coexist, as predicted by our theoretical framework. In the ordinate axis use the notation $\bar{\eta }$ and not *η*.

Therefore, within our framework, we can analytically study the impact of the species interaction network architecture on system stability and species extinction. Two simple examples are shown corresponding to an ecosystem with no extinction (see Fig. 3A,B) and with extinction (see Fig. 3C,D). Additionally, a real-world example is also shown (see Fig. 3E,F).

**Figure 4:**
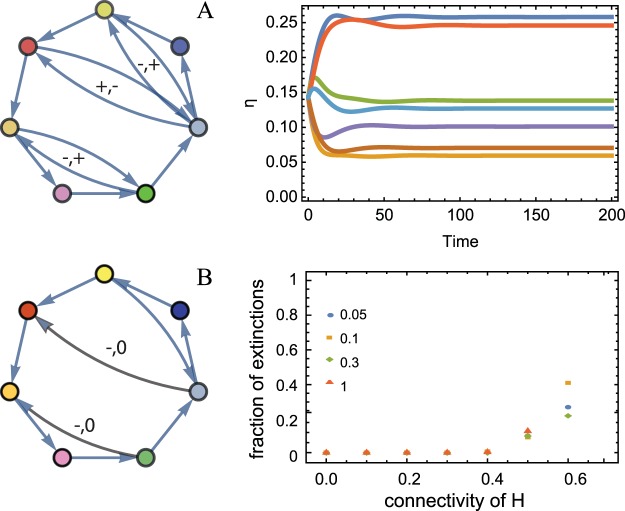
The fraction of extinctions of network with exploitative or harmful interactions as well as cooperative interactions. (**A**) Species interaction network for 7 species where each species *i* has one mutualistic partner *j*, i.e. *M*_*ij*_ = 1, some species have exploitative interactions (+ −) and cooperative and exploitative intensities are ${\epsilon }_{1}$ = ${\epsilon }_{2}$ = 1. The corresponding time evolution of the populations of the 7 species, as predicted by the mean field dynamics Eq. (2), are also shown. During the time evolution, the rates given by Eq. (1) remain positive and extinctions are not observed. (**B**) Example of a network with harmful interactions and plot of the fraction of extinctions (defined as the fraction of species extinct in the community) as a function of the connectivity of harmful matrix *H* (*c*_*H*_) and with connectivity of the cooperative matrix *M* (*c*_*M*_ = 1 − *c*_*H*_) for different average interaction strengths (colored points) *μ*_*M*_ = −*μ*_*H*_ = 0.05, 0.1, 0.3, 1 (see legend) and cooperative and harmful intensities are ${\epsilon }_{1}$ = ${\epsilon }_{H}$ = 1. The off-diagonal elements of matrices *M* and *H* are drawn from normal distribution, i.e. *M*_*ij*_ ~ *z*, *H*_*ij*_, *H*_*ji*_ ~ 0, −*z*, *z* ~ N (*μ*_*M*_, *μ*_*M*_/3). The network size considered in these simulations is *S* = 50 and each point is the mean of 10 realizations. Similar results are found also for *S* = 20, *S* = 100 (not shown here).

We then turn on also exploitative interactions (${\epsilon }_{2}$ > 0) and we numerically find that, even in presence of relatively large concentrations of exploitative interactions (*c*_*L*_), at stationarity the system still admits an high biodiversity and full coexistence is observed, as long as a mutualistic network of interactions is present, corresponding to an irreducible matrix, *M* (see Fig. 4A and Methods). Further discussion can be found in the Supplementary Information, Section S4.

### Effect of harmful interactions

The current model can also take into account the harmful interactions such as impact of noxious secreted compounds (e.g. antibiotics and fosfomycin molecule) on species biodiversity. We can thus introduce a further directed network *H* on the set {1, …, *S*}, where *H*_*ij*_ representing indirect harmful relationships (−0), i.e. a directed link of strength *H*_*ij*_ from *i* to *j* means that the *j*-th species receives a harmful effect from the *i*-th species. The overall species interaction network is defined through three matrices *M*, *L* and *H* where *M*_*ij*_ representing cooperation interactions (+ +, + 0), *L*_*ij*_ representing exploitation interactions (+ −) and *H*_*ij*_ representing harmful interactions (−0). These matrices satisfy the following conditions for all *i*, *j* = 1, …, *S*: (i) *M*_*ij*_ ≥ 0. A directed link of strength *M*_*ij*_ from *i* to *j* means that the *j*-th species receives a beneficial effect from the interaction with the *i*-th species; (ii) *L*_*ij*_*L*_*ji*_ < 0 or *L*_*ij*_ = *L*_*ji*_ = 0. *L*_*ij*_ > (<0) and *L*_*ji*_ < 0(>0) denotes that the *j*-th species exploits (is exploited by) the *i*-th species; (iii) *H*_*ij*_ < 0, *H*_*ji*_ = 0 or *H*_*ij*_ = 0, *H*_*ji*_ < 0 or *H*_*ij*_ = *H*_*ji*_ = 0. A directed link of strength *H*_*ij*_ from *i* to *j* means that the *j*-th species receives a harmful effect from the interaction with the *i*-th species; (iv) *L*_*ij*_*M*_*ij*_*H*_*ij*_ = 0 that is each pair of species, *i* and *j*, can have at most one of the above interactions.

The term of harmful interactions is linear in $\bar{\eta }$ (i.e. ${\bar{\eta }}^{k}{H}_{kj}$), so the dynamics as induced by the Markovian evolution on the relative abundance ${\bar{\eta }}^{s}$ of each species lead to the following mean field equation:

3 $$\begin{array}{rcl}\frac{{\rm{d}}}{{\rm{d}}t}{\bar{\eta }}^{s}(t) & = & {\epsilon }_{1}\sum _{k=1}^{S}{\bar{\eta }}^{k}(t){M}_{ks}\theta ({\bar{\eta }}^{s}(t))+{\epsilon }_{2}\sum _{k=1}^{S}{\bar{\eta }}^{k}(t){L}_{ks}{\bar{\eta }}^{s}(t)+{\epsilon }_{3}\sum _{k=1}^{S}{\bar{\eta }}^{k}(t){H}_{ks}\theta ({\bar{\eta }}^{s}(t))\\  &  & -\,\,{\bar{\eta }}^{s}(t)\sum _{i,k=1}^{S}({\epsilon }_{1}{\bar{\eta }}^{k}(t){M}_{ki}\theta ({\bar{\eta }}^{i}(t))+{\epsilon }_{2}{\bar{\eta }}^{k}(t){L}_{ki}{\bar{\eta }}^{i}(t)+{\epsilon }_{3}{\bar{\eta }}^{k}(t){H}_{ki}\theta ({\bar{\eta }}^{i}(t)))\end{array}$$

for *s* = 1, …, *S* where $\sum _{j=1}^{S}{\bar{\eta }}^{j}(t)=1$ and is conserved by the dynamics.

For simplicity but without loss of generality, we turn off the exploitations (given by the *L* matrix) and thus consider only the interaction matrices *M* and *H*, i.e. we focus on cooperation and on the effects harmful interactions mediated by secreted compounds. We discuss the results for a fully connected network, *c*_*M*_ + *c*_*H*_ < 1, since the case *c*_*M*_ + *c*_*H*_ < 1 does not alter our main conclusions. Of course, we only consider feasible and stable solutions. To do that, we first find the stationary solutions and then calculate the Jacobian matrix evaluated at that solution to check the stability. Figure 4B shows the fraction of extinction as a function of *c*_*H*_. We consider an extinction to occur if the species abundance goes below an extinction threshold 1/*S*^2^, where *S* is the number of species (notice that the average population density in the uniform case is 1/S). When *c*_*H*_ > 0 the PF theorem cannot be applied. However, we start to detect extinctions only after a critical threshold of harmful interactions in the system and the extinction rate is an increasing function of *c*_*H*_. For *c*_*H*_ larger than 0.6, the only feasible solutions are not stable and thus the extinction rate is not shown. Therefore, as expected, the existence of such molecules challenges the unlimited coexistence seen in the purely cooperative model.

### Cooperation determines ecosystem stability

**Figure 5:**
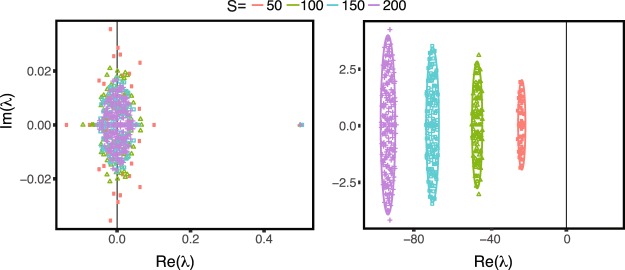
Eigenvalues spectrum of the Jacobian matrix *J*. (**A**) Pure exploitative interactions where *C*_*L*_ = 1, ${\epsilon }_{2}$ = 1 and ${\epsilon }_{1}$ = 0. (**B**) Exploitative and mutualistic interactions where *C*_*L*_ = *C*_*M*_ = 0.5, ${\epsilon }_{1}$ = 0.75 and ${\epsilon }_{2}$ = 1. The off-diagonal elements of matrices *M* and *L* are drawn uniformly between 0 and 1, i.e. *M*_*ij*_ ~ *z*, *L*_*ij*_, *L*_*ji*_ ~ ±*z*, *z* ~ *U*(0, 1). The points are the eigenvalues of one Jacobian matrix obtained sampling at random the matrices *M* and *L*, while the lines indicate the analytical prediction for the support of the *J* eigenvalues in the corresponding cases (see Eq. (4)). The black vertical line indicates the instability threshold.

We now study analytically the stability of the equilibria for an ecosystem with cooperative and exploitative interactions, by analyzing the eigenvalues of the linearization of Eq. (2), i.e. the Jacobian matrix *J*, around the equilibria, *m*_*i*_, as a function of ecological complexity (*c* × *S*). We set the diagonal elements of *M* to zero and the off-diagonal pair (*M*_*ij*_, *M*_*ji*_) is equal to (0, 0) with probability 1 − *c*_*M*_ and with probability *c*_*M*_ drawn from a bivariate Gaussian distribution of means (*μ*, *μ*)^T^ and covariance matrix ${\rm{\Sigma }}=({\sigma }^{2},\rho {\sigma }^{2};\rho {\sigma }^{2},{\sigma }^{2})$. This guarantees that, for a connected cluster, coexistence of all species occurs. The mean, variance and correlation of the non-diagonal elements of matrix *M* are ${\mu }_{L}={c}_{L}\mu$, ${\sigma }_{M}^{2}={c}_{M}{\sigma }^{2}+{c}_{M}(1-{c}_{M}){\mu }^{2}$ and ${\rho }_{M}=\frac{\rho {\sigma }^{2}+(1-{c}_{M}){\mu }^{2}}{{\sigma }^{2}+(1-{c}_{M}){\mu }^{2}}$. The case in which each element of *M*_*ij*_ is assigned independently of *M*_*ji*_ simply corresponds to the case *ρ* = 0 (notice that even if *ρ* = 0 we can have *ρ* ≠ 0). The exploitative matrix *L* is considered similarly. If ${\mu }_{M}\ge {\sigma }_{M}\sqrt{(1+{\rho }_{M})/S}$, the leading eigenvalue ${\lambda }_{M}=S{\mu }_{M}=S{c}_{M}\mu$ and the corresponding eigenvector has positive components^14^. Moreover, the components of the leading eigenvector are approximately constant, i.e. the equilibria of system given by Eq. (2) can be written as ${m}_{i}=(1+{\xi }_{i})/S$ for *i* = 1, …, *S* with $\sum _{i}{\xi }_{i}=0$. Using the fact that $1=S{m}_{i}-{\xi }_{i}$, ${\lambda }_{M}=S{\mu }_{M}$ and taking into account that all the terms involving *ξ*_*j*_ are sub-leading in *S*, we obtain that the leading term of the system Jacobian does not depend on *L* (see Methods) and it is equal to:

4 $${J}_{ij}=-\,{\delta }_{ij}S{\mu }_{M}+({M}_{ij}-{\mu }_{M})=-\,{\delta }_{ij}S{\mu }_{M}+{M^{\prime} }_{ij}$$

where ${M^{\prime} }_{ij}\,:\,={M}_{ij}-{\mu }_{M}$ is a random matrix with zero mean, variance ${\sigma }_{M}^{2}$ and correlation *ρ*_*M*_. This implies that the eigenvalues are uniformly distributed in an ellipse centered around −*Sμ*_*M*_ with semi-axis $\sqrt{S}{\sigma }_{M}(1+{\rho }_{M})$ and $\sqrt{S}{\sigma }_{M}(1-{\rho }_{M})$^14^. The largest eigenvalue of the Jacobian is therefore given by $-S{\mu }_{M}+\sqrt{S}{\sigma }_{M}(1+{\rho }_{M})$. Thus, for fixed connectivity, *c*, in the presence of cooperation the system stability increases with *S*, where as if only predator-prey interactions are present, then the system is always unstable for large enough *S*, as the May theorem would predict (see Fig. 5 and Supplementary Information, Section S5).

## Discussion

In this work we have proposed how to incorporate the effect of indirect cooperation induced by habitat modification or cross-feeding^19,27,37^ in order to have effective equations where resources are not explicitly modelled (as in Lotka Volterra equations). This framework is a possible choice to model species dynamics with implicit resources and cooperative interactions. The introduction of indirect mutualism changes the whole stability profile of the corresponding system’s dynamics. This result highlights the importance of having a microscopic derivation of the phenomenological models and the birth rate should be accurately chosen depending on the specific ecological system one wants to model. Our results can be applied to study the effect of the interaction network topology on species coexistence in real ecological communities. In particular, we found that nested architecture, observed in plant-pollinators ecological communities^15,18^, where specialist species, with only few mutualistic links, tend to interact with a proper subset of the many mutualistic partners of any of the generalist species, (see Fig. 3E) satisfies the hypothesis of the PF theorem and thus favor species coexistence (see Fig. 3F). We have then shown that by properly deriving the contribution of cooperation in the species population dynamics, we solve two long-standing problems in theoretical ecology: how a large number of species can coexist together and the complexity-stability paradox. In fact, we found that cooperation promotes ecosystem biodiversity, that in turn increases its stability without any fine-tuning of the species interaction strengths or of the self-interactions. Even moderate cooperative interactions can stabilize the dynamics and the stability increases with the ecosystem complexity (see Fig. 5). In our framework we can also model and studies the effect of harmful secreted compound, such as antibiotics. We observe that these harmful interactions may lead to the extinctions of targeted species, but also in this case our conclusions still hold: cooperation promotes ecosystem biodiversity and stability. Of course, if we add direct cooperation beside the mediated mutualism, such as classic Holling quadratic interactions term, as long as indirect linear mutualism is present, we observe violation of the classic complexity-stability paradox (see Supplementary Information, Section S6).

Our results can be compared to the ones obtained recently by Servan *et al*.^9^, where it was shown that in random Lotka-Volterra systems many species were coexisting. It is important to underline, that that result was obtained assuming the stability of the interaction matrix, as the focus was to study the effect of feasibility. If the interaction matrix is not stable, in fact, one should expect more extinctions^10^. Conversely, in this work, a high level of coexistence is obtained without assuming the stability of the interaction matrix or, equivalently, small interaction strengths. All presented results consider effective competition by fixing the system carrying capacity, i.e. an organism comes into the system always at the expenses of another organism. The results do not change when the hard constraint of total fixed population size is relaxed by introducing the possibility for a site to become empty (see Supplementary Information, Section S7).

Finally, we may also consider a constant effort hypothesis^38^ in regulating species interactions. This would translate in normalizing the strengths of each interaction matrix by the by corresponding species in-degree (see Supplementary Information, Section S8). Nevertheless, in all these different scenarios our conclusions still hold: we find that a shift in the assumptions behind cooperative interactions resolve long-standing open theoretical question on the relation between stability and complexity and provides a unifying modeling approach to describe emergent patterns in ecology and interacting large ecological systems, as observed recently in real microbial communities^27,37^.

## Methods

### Application of the Perron-Frobenius theorem to the model equations

Let us consider the dynamics given by Eq. (2) for ${\epsilon }_{2}$ = 0. If *M* is irreducible, then the PF theorem holds^36^ and given the initial condition ${\bar{\eta }}^{s}(0) > 0$ where *s* = 1, …, *S*, the time dependent solution for the species fractions is

5 $$\bar{\eta }(t)=\frac{\bar{\eta }{(0)}^{T}{e}^{\epsilon Mt}}{\sum _{s}{(\bar{\eta }{(0)}^{T}{e}^{\epsilon Mt})}_{s}}$$

Since for any eigenvalue, *β* ≠ *α*, of *M* we have $\Re (\beta ) < \alpha$, the dominant term in both numerator and denominator in Eq. (5) is $v\,{e}^{\alpha t}(\bar{\eta }(0)\cdot w)$ leading to $\mathop{\mathrm{lim}}\limits_{t\to \infty }\bar{\eta }(t)=\frac{v}{\sum _{s}{v}_{s}}=m$. This is an easy computation when *M* has a basis of eigenvectors and in general can be derived using the Jordan decomposition. As a corollary of the derivation above, we have also that the stationary solution is globally stable in the region ${\bar{\eta }}^{s}(0) > 0$ for all *s* = 1, …, *S*.

### Analytical justification of the coexistence condition in the presence of exploitative interactions

As explained in the main text, if the matrix *M* is irreducible and the transition rates given by Eq. (1) are positive during the time evolution (a necessary condition in order that the derivation of the mean field is justified), then we find numerically that, even in presence of a large concentrations of exploitative interactions, at stationarity the system still admits an high biodiversity and full coexistence is observed. Here we want to heuristically justify what we have observed numerically. Adding exploitative interactions does not lead to extinction, as long as the mutualistic network of interactions is present, corresponding to an irreducible matrix, *M*. We argue that, under this hypothesis, when ${\bar{\eta }}^{s}$ is positive but close to zero the complete mean field equations - where both ò_1_ and ò_2_ are positive - are perturbation of the mean field equation where only mutualistic interaction are present, since we have proved that a pure mutualistic system has no extinction as long as the matrix *M* is irreducible. Following the notation in Results, our continuous time Markov process is defined by the rule: a randomly chosen individual is removed and substituted by an individual of the *j*-th species at a rate

6 $$\omega (j,\eta ,M,L)=\mathop{\underbrace{{\bar{\eta }}^{j}+{\epsilon }_{1}\sum _{k=1}^{S}{\bar{\eta }}^{k}{M}_{kj}\theta ({\bar{\eta }}^{j})}}\limits_{:={\omega }_{j}^{M}}+\mathop{\underbrace{{\epsilon }_{2}\sum _{k=1}^{S}{\bar{\eta }}^{k}{L}_{kj}{\bar{\eta }}^{j}}}\limits_{:={\omega }_{j}^{L}}$$

where ${\epsilon }_{1}$ > 0 and ${\epsilon }_{2}$ > 0 give the cooperation and exploitation intensity, and $\theta (\,\cdot \,)$ is the Heaviside step function, i.e., *θ*(*x*) > 0 when *x* > 0 and 0 otherwise. As *N* → ∞ the relative abundance ${\bar{\eta }}^{s}$ converges to the solution of the system of ordinary differential equation for *s* = 1, …, *S*. Equation for ${\bar{\eta }}^{s}$, when ${\bar{\eta }}^{s}$ is positive but close to zero, can be written in the following form

7 $$\frac{{\rm{d}}}{{\rm{d}}t}{\bar{\eta }}^{s}(t)=\mathop{\underbrace{{\omega }_{s}^{M}-{\bar{\eta }}^{s}(t)\sum _{i=1}^{S}{\omega }_{i}^{M}}}\limits_{\simeq \delta  > 0}+{\mathscr{O}}({\bar{\eta }}^{s})$$

The first two terms in Eq. (7) are the vector fields corresponding to mean field equation for *M* irreducible and no exploitation (i.e. ${\epsilon }_{2}$ = 0). We know that such a system has no extinction and its vector field is typically greater than *δ* > 0 out of equilibrium when ${\bar{\eta }}^{s}\simeq 0$. The last term in Eq. (7) contains terms which are linear dependent of ${\omega }_{j}^{L}$ which is ${\mathscr{O}}({\bar{\eta }}_{s})$. Thus $\frac{{\rm{d}}}{{\rm{d}}t}{\bar{\eta }}^{s}(t)$ is positive for ${\bar{\eta }}^{s}$ close to zero. The requested transition rates never become negative during the time evolution of the mean field equation. This is a necessary condition otherwise the derivation itself of the mean filed equation would be meaningless.

### Stability of the equilibria

In the case of ${\epsilon }_{2}$ ≠ 0, the entries of the Jacobian read

8 $$\begin{array}{rcl}{J}_{ij} & = & {\epsilon }_{1}({M}_{ij}^{T}-{\delta }_{ij}\sum _{h,k=1}^{S}{m}_{h}{M}_{hk}-{m}_{i}\sum _{k=1}^{S}{M}_{jk})+{\epsilon }_{2}({L}_{ji}{m}_{i}+{\delta }_{ij}\sum _{h=1}^{S}{m}_{h}{L}_{hi}-{\delta }_{ij}\sum _{h,k=1}^{S}{m}_{h}{L}_{hk}{m}_{k}\\  &  & -\,{m}_{i}\sum _{k=1}^{S}{L}_{jk}{m}_{k}-{m}_{i}\sum _{k=1}^{S}{L}_{kj}{m}_{k})\end{array}$$

The diagonal entries of the Jacobian are

9 $${J}_{ii}=-\,{\epsilon }_{1}\sum _{h,\,k=1}^{S}{m}_{h}{M}_{hk}+{\epsilon }_{2}\sum _{h=1}^{S}{m}_{h}{L}_{hi}-{\epsilon }_{2}\sum _{h,\,k=1}^{S}{m}_{h}{L}_{hk}{m}_{k}$$

Since ${m}_{i} \sim 1/S$, it is simple to observe that the term proportional to ${\epsilon }_{1}$ is of order *S* (plus sub-leading fluctuations). On the other hand, the leading order of the terms proportional to ${\epsilon }_{2}$, is of order 1 and therefore always sub-leading if ${\epsilon }_{1}$ > 0. A similar argument applies to the off-diagonal elements. In that case, the terms proportional to ${\epsilon }_{2}$ are of order 1, while the ones proportional to ${\epsilon }_{2}$ are of order 1/*S*.

Similarly to what found in the case ${\epsilon }_{2}$ = 0, we have that the following relations hold: *μ*_*L*_ = *C*_*L*_*μ*, ${\sigma }_{L}=\sqrt{{c}_{L}({\sigma }^{2}+(1-{c}_{L}){\mu }^{2})}$ and ${\rho }_{L}=\frac{\rho {\sigma }^{2}+(1-{c}_{L}){\mu }^{2}}{{\sigma }^{2}+(1-{c}_{L}){\mu }^{2}}$ where *μ* and *σ* are the mean and the standard deviation of the distribution from which we draw the value for the exploitative interaction strengths. These expressions have been used together with *μ*_*M*_, *σ*_*M*_ and *ρ*_M_, when calculating the coefficient of variation. The above considerations indicate that the distribution of the eigenvalues of the Jacobian, Eq. (9), is the same as the ${\epsilon }_{2}$ = 0 case. Figures visualizing these results are presented in the Supplementary Information, Section S5.

## Supplementary information

Supplementary Information
