# Supplementary material for: Reconciling cooperation, biodiversity and stability in complex ecological communities
Source: Sci Rep. 2019 Apr 3;9:5580. doi: 10.1038/s41598-019-41614-2 (PMC6447617; doi:10.1038/s41598-019-41614-2)
Supplement: Supplementary file 1 — Supplementary Information [file 41598_2019_41614_MOESM1_ESM.docx]

**Reconciling cooperation, biodiversity and stability in complex ecological communities (Supplementary Information)**

Chengyi Tu^1,2^†, Samir Suweis^3^†*, Jacopo Grilli^4,5^, Marco Formentin^6^*, Amos Maritan^3^*

^1^School of Ecology and Environmental Science, Yunnan University, 650091, Kunming, China. ^2^Department of Environmental Science, Policy, and Management, University of California, Berkeley, CA 94720-3114, USA. ^3^Department of Physics and Astronomy “Galielo Galilei”, University of Padova, Via Marzolo 8, 35131 Padova, Italy. ^4^Santa Fe Institute, 1399 Hyde Park Road, Santa Fe, NM 87501, USA. ^5^The Abdus Salam International Centre for Theoretical Physics (ICTP), Strada Costiera 11, 34014 Trieste, Italy. ^6^Department of Mathematics “Tullio Levi-Civita”, University of Padova, Via Trieste 63, 35129 Padova, Italy.

†These authors contributed equally to this work.

*Correspondence and requests for materials should be addressed to S.S. (email: [suweis@pd.infn.it](mailto:suweis@pd.infn.it)), M. F. (email: marco.formentin@unipd.it) and A. M. (email: maritan@pd.infn.it)

**S1. Mechanistic interpretation of linear growth rates**

The origin of the linear form in mediated cooperative interactions (mutualism or commensalism) could be justified in various ways, as explained in the main text. A first mechanism of indirect cooperation is habit modification, i.e. a species modifies the surrounding environment in a way that favors other species. Another possible source of mediated cooperation is when inter-specific cross-feeding is present. Effects of resource exchange have been recently studied in many experiments^1-5^, providing evidences that it enhances biodiversity and stability of microbial communities (contrary to previous expectations). Therefore, we here provide details on how our theoretical framework may be useful to understand and interpret these recent findings. If a species secretes chemical compounds that are favorable for more than one species, say $k$ species, the hypothesis under which this leads to the linear interaction term is when the nourishment is used by each of the $k$ species at different time. Thus, in a given period of time all $k$ species can profit of the nourishment but they are not doing so simultaneously. In fact, microbial species typically display a preference in the order by which different substrates (for which it possesses enzymatic proficiency) are actually consumed^6^. As a classic example, when two sugars as glucose and lactose are both present, Escherichia coli will first consume all the glucose and only afterwards it will start degrading lactose^7^. Likewise, in the presence of different metabolites secreted by species A, and that could be exploited by more than one other species, the time at which species B, C, D etc. will access each given compound will depend on: (i) their diauxic preference order towards the series of substrates; (ii) the presence or absence of the corresponding degradative enzymes in their genotypes. The following example will clarify this simple scenario.

Assume that (1) compounds ‘x’ and ‘y’ are released by species A; (2) Species B lacks the functional gene for the catabolic enzyme required to use ‘x’ and it will only be able to consume ‘y’; (3) Species C can consume both but it has a diauxic preference to use ‘x’ first. It ensures that during an interval of time $=T_{1}$ species B will consume ‘y’ while species C will consume ‘x’: subsequently at a later interval of time $=T_{2}$ (non-overlapped with the previous one) species C will consume ‘y’. Compound ‘y’ is therefore used by different species during different temporal windows.

The explicitly mathematical explanation is as following: The state at time $t$ of the system is given by species concentration vector (i.e., average fraction of individuals for each species) $\bar{\eta}^{i}(t)$ with $i=1,\ldots,S$ and let $c$ be the concentration of a given resource used by the species $j$. This resource is provided, at a rate $s$, by certain species, $k$ (e.g. through metabolic waste/secretion^5,8^ or, in the case of plant/flowers, it represents the pollen produced by the $k$’s species) and related to their populations in a linear way, that is $s(t)=\sum_{k} \bar{\eta}^{k}(t)M_{kj}$. Let us assume first that a single species, say the $j$-th one, is consuming this resource. The kinetic of nutrient concentration is then

$$\frac{dc(t)}{dt}=\frac{1}{\tau_{R}}\left(s(t)-\bar{\eta}^{j}(t)r(c(t))\right)$$

where $r(c)$ is the consumption rate per individual whose specific form is irrelevant for the purpose of this example (e.g., one can consider the Monod function $r(c)=\alpha c/(K+c)$, with $\alpha$ and $K$ some suitable constants). The constant $\tau_{R}$ is the timescale of the dynamics of resources.

The contribution to the growth rate $\Delta\omega_{j}$ of the $j$-th species, due to this nutrient, is

where is a conversion factor measuring how the nutrient contributes to the biomass of the $j$-th species. An assumption that is generally made when treating consumer-resource models is that resource dynamics is much faster than population dynamics: metabolic processes are generally order of magnitudes faster than reproductive ones, so this can be a reasonable approximation. In practice this means that the resources' abundances immediately reach their steady-state values, so we can set in Eq. and express $r_{i}$ as a function of $n_{\sigma}$^9^:

$$r(c(t))=\frac{s(t)}{\bar{\eta}^{j}(t)}$$

leading to

If $\bar{\eta}^{j}>0$, then we have that the rate is linear in the population of the mutualistic partners. If many species use the same resource we assume, according to the above discussion, that they do that at different non-overlapping time intervals. This is implemented in terms of a matrix $M(t)$ whose time dependence is such that at each time $t$ and for each species $k$ at most another species, $j$, exists such that $M_{kj}(t)>0$ and $M_{km}(t)=0$ if $m\neq j$. In a given period of time, $\tau$, all species using the same resource are fed. The mean field equation, Eq. (2), in the main text still holds with $M(t)$. Eq. and our analysis in the main text correspond to approximate the dynamics with $M$ as given by the time average of $M(t)$, i.e. $M=\int_{0}^{\tau} M(t)dt/\tau$. When the results concerning the existence and the approach to the stationary state, given in Materials and Methods and the corresponding discussion in the main text remain valid with substituted by $(U^{T})^{k}$ for a time $t$ equal to an integer number, $k$, of periods $\tau$ where ( is the time ordering operator). If the time average of $M(t)$, $\int_{0}^{\tau} M(t)dt/\tau$, is irreducible, it implies that also $U$ is irreducible. Thus also in this case we can apply the Perron-Frobenius theorem and get that $\lim_{k\to\infty} \bar{\eta}(k\tau)=m$ where $m$ is the right eigenvector of $U$ such that $\sum_{i} m_{i}=1, m_{i}>0$, corresponding to the unique positive eigenvalue larger than the modulus of all other eigenvalues of $U$^10^. The latter assumption is in fact only appropriate when we are assuming that the contribution to the population growth depend on the physical encounter between two species, as typically happens for exploitative (e.g. predator-prey) interactions.

We should have emphasized that in our mean field equations the dynamical variables, the species populations (or the fraction of them), are treated as continuous variables, as it is commonly done in many approaches besides to the ecological context (e.g. condensed matter, statistical mechanics etc.). This is a reasonable approximation when the species’ populations correspond to a non-vanishing fraction of the total population and in the limit of large total population. Moreover, the above mechanisms only hold if species densities are not too small, otherwise other biological limitations to growth come into play. Thus, our mean field, as many other approaches using continuous variables as approximation of discrete ones, cannot afford to capture the limit case^11^ of very low density.

**S2. Relative species abundance, covariance matrix and species interaction matrix**

The cumulative RSA is thus defined as the fraction of species with population greater that a certain value $n$, $P_{>}[n]=\frac{1}{S}\sum_{k=1}^{S} \theta(n-Nm_{k})$, where we have fixed $N=1/\min\{m_{1},\ldots,m_{S}\}$ when all species coexist, i.e. we have made the choice that the rarest species has population equal to 1. Here we only consider cooperative and exploitative interactions. We numerically find that the stationary RSA displays a log-normal shape, and it is mainly determined only on its coefficient of variation, $\text{CV}=\sigma_{M+L}/\mu_{M+L}=(\sqrt{\sigma_{M}^{2}+\sigma_{L}^{2}})/(\mu_{M}+\mu_{L})$. If the diagonal elements of cooperative matrix $M$ are zeros and the off-diagonal pair $(M_{ij},M_{ji})$ is equal to $(0,0)$ with probability $1-c_{M}$ and drawn from a bivariate Gaussian distribution of means $(\mu,\mu)^{T}$ and covariance matrix $\Sigma=(\sigma^{2},\rho\sigma^{2};\rho\sigma^{2},\sigma^{2})$ with probability $c_{M}$, then we have mean $\mu_{M}=c_{M}\mu$, variance $\sigma_{M}^{2}=c_{M}\sigma^{2}+c_{M}(1-c_{M})\mu^{2}$ and correlation $\rho_{M}=\frac{\rho\sigma^{2}+(1-c_{M})\mu^{2}}{\sigma^{2}+(1-c_{M})\mu^{2}}$ (also explained in Methods, Stability of the equilibria). The exploitative matrix $L$ is considered similarly. Therefore, putting together these expressions, we obtain the analytical formula for the CV of the matrix $A=M+L$.

We now consider the normal fluctuations around the deterministic limit of Eq. (2) in the main text. This allows us to calculate the matrix $V$ describing the correlation between pairs of species population abundances. As highlighted in the main text, this quantity, once the threshold is set opportunely, is used as an empirical proxy of the species interactions network^12,13^. Other works, applying maximum entropy approach, use $V^{-1}$ as the quantity to describe species interactions^14^. The aim of this section is to test how well $V$ or $V^{-1}$ approximates to the true interactions described by $M+L$ in our model.

For sake of simplicity, we assume that the limiting dynamics start at the equilibrium $m_{1},\ldots,m_{S}$ with $0<m_{i}<1$ where $i=1,\ldots,S$. Thus, we define the fluctuation process as

$$x_{N}^{i}(t)=\sqrt{N}\left(\bar{\eta}_{N}^{i}(t)-m_{i}\right)\text{ for }i=1,\ldots,S$$

One can apply standard techniques of convergence of generators to get weak convergence to the thermodynamic limiting evolution ^15^. Indeed, the stochastic process $\left(x_{N}^{1}(t),\ldots,x_{N}^{S}(t)\right)$ converges in distribution to a Gaussian Markov process $X:=\left(X^{1}(t),\ldots,X^{S}(t)\right)$ which solves the stochastic differential equation

$$dX= AX dt+\Phi dB_{t}$$

where $B_{t}$ is a $S$-dimensional Brownian motion and

where $i, j=1,\ldots,S$ and $\delta_{ij}$ is the Kronecker delta.

From Eq. , it is then possible to derive the dynamics of the covariance matrix (see^16^ for details):

$$V_{ij}(t)=\langle X^{i}(t)X^{j}(t)\rangle-\langle X^{i}(t)\rangle\langle X^{j}(t)\rangle$$

Therefore, we have

$$\frac{d V(t)}{dt}= A V(t)+ V(t) A^{T}+\Phi\Phi^{T}$$

and at stationarity the covariance matrix, $V_{ij}=\lim_{t\to\infty} V_{ij}(t)$, resolves the following equation

$$A V+ V A^{T}+\Phi\Phi^{T}=0$$

Eq. is a Lyapunov equation, so we could apply standard algorithms to solve it numerically^17^.

We have determined $V$ from the solution of Eq. and then determined $V^{-1}$. If one assumes that the population fluctuations around their means are Gaussian distribution, then $V^{-1}$ represents the species interaction matrix ^14^. In our framework and as shown by Eq. and , the relation between the interaction matrix $M+L$ and the matrix $V$ or $V^{-1}$ is highly non-linear. Moreover, because of the constraint, $\sum_{j} V_{ij}=0$, $V$ is not invertible, and thus in order to compute $V^{-1}$ we apply a pseudo-inverse scheme, i.e. we invert $V$ by the subspace of spanned by the eigenvectors corresponding to non-zero eigenvalues. As shown in Fig. S1, even for very simple structure of matrix $M$ and $L$, $V$ and $V^{-1}$ are not good proxies of the species interactions. The results are shown for the model without empty sites, but there is no qualitatively difference with the model including empty sites. This result highlights the importance to properly infer interaction networks from data.


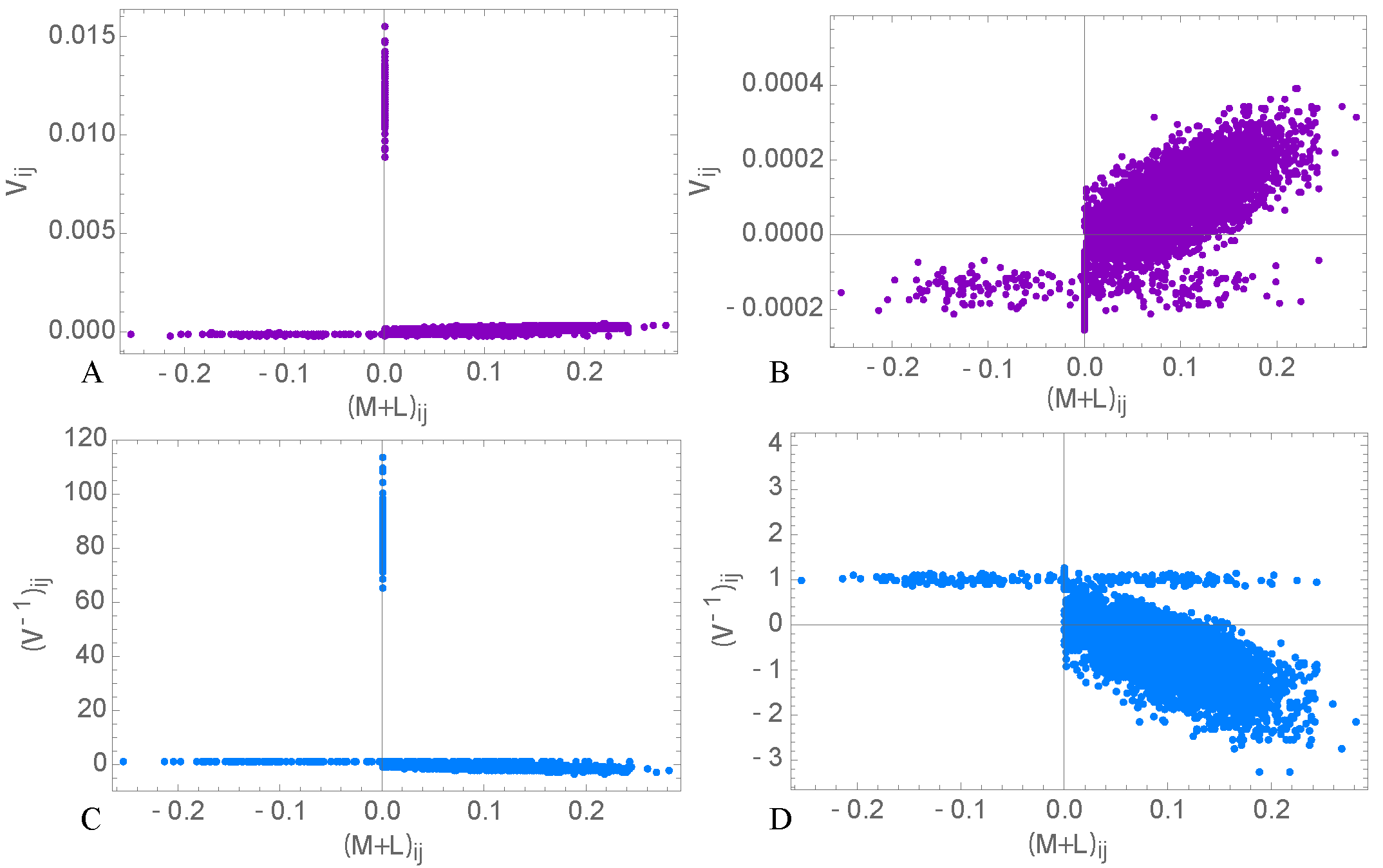


**Fig. S1. Elements of the covariance matrix $V$ and its inverse $V^{-1}$ compared to the interaction matrix $M+L$.** **(A)** The correlation between $M+L$ and $V$. **(B)** Its zoom in the relation closes to the intersection. **(C)** The correlation between $M+L$ and $V^{-1}$. **(D)** Its zoom in the relation closes to the intersection. Matrix size $S=100$, dense mutualism $C_{M}=0.5$ and sparse exploitation $C_{L}=0.05$. Interaction strengths $z_{ij}$ have been drawn from a Gaussian distribution of mean $\mu_{N}=0.1$ and standard deviation $\sigma_{N}=0.05$. The sign has been then chosen accordingly ($M_{ij}=|z_{ij}|$, $L_{ij}=|z_{ij}|$ and $L_{ji}=-|z_{ji}|$). We have also imposed the irreducibility of $M$. Although their zooms highlight a slightly positive or negative correlation between elements of $M+L$ and $V$ or $V^{-1}$ respectively, they are not significant. Most of the elements of both the covariance matrix $V$ and its inverse $V^{-1}$ are close to zero. Other elements are very large, although the corresponding species do not interact, indicating that $V$ or $V^{-1}$ cannot be used as interaction matrix.

**S3. Topology of the interaction networks, coexistence and stationary states**

In this section, we discuss some features of the topology of the cooperative matrix $M$ and how they relate to stationary states of the system. Indeed, some properties of the large volume dynamics of our model can be read from the topology of the interaction matrix $M$. We recall the matrix $M$ is not the transition matrix of the Markovian dynamics, instead it describes the network topology of (cooperative) interactions among species of an ecosystem. The main concept in this section is pruned network and the operation of pruning a network. A node with in-degree equals to zero and out-degree different from zero is called a dead leaf of the network. The operation of pruning consists in eliminating one by one the dead leaves of a given network together with their outbound links. After a first pruning, we will obtain a new network (that is a subnetwork of the starting one) that may still have dead leaves - the elimination of dead leaves may create new dead leaves. The pruning process ends when the resulting network has no more dead leaves. The latter network is called stable or pruned. It is easy to see that the minimal pruned network (i.e. with the smallest number of links) that can be constructed with $S$ nodes is the cyclic network. More in general, we have: The pruned network is a union of isolated nodes and networks that contain at least one cycle each.

Indeed, pruning stops when the obtained network is a union of isolated nodes and networks where all nodes have at least an ancestor (i.e. the in-degree of each node is positive). Now a finite network where each node has a least one incoming link contains at least a cycle. In fact, starting from one node it is possible to walk through the ancestors and never stop. Since the network is finite, soon or later, the walker will visit twice the same node - so the walk contains a cycle - at most after a number of steps that equal the size of the network.

The pruned network has at least one cycle but when not simply union of isolated cycles it can be very complex. Fig. S2 shows an example of the pruning process and of a non-trivial pruned network.


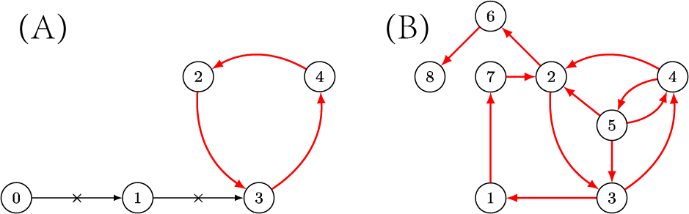


**Fig. S2. Diagram of how to prune network. (A)** An example of the pruning process. First the 0-node is eliminated with its outbound link. After that, the node 1 becomes a dead leaf and has to be pruned. The cycle shown by the red links is the resulting pruned network. **(B)** An example of a pruned network that is not composed only by cycles.

As we anticipated at the beginning of this section, the dynamics of species sitting on dead leaves of the interaction network are trivial as their relative abundance goes to zero. This is a simple consequence of the fact that a dead leaf has no incoming bond. Thus, when $s$ is a dead leaf, the first term on the right of Eq. (2) in the main text is zero and simple estimate gives . The previous simple remark leads to the following:

*Limiting dynamics of dead leaves*: Start the dynamics from a point with $\bar{\eta}^{i}\neq0$ for all $i=1,\ldots,S$. If $k$ is a dead leaf then $\lim_{t\to\infty} \bar{\eta}^{k}(t)=0$.

Thus, the presence of a dead leaf inhibits coexistence equilibria on the whole network. More precisely, if $i=1,\ldots,\gamma$ are dead leaves (at some step of the pruning), the stable equilibria must have $\bar{\eta}^{1}=\ldots=\bar{\eta}^{\gamma}=0$.

**S4. Mean field equations, birth rates and species coexistence**

We have numerically and systematically investigated the number of extinctions in ecological systems with both cooperative and exploitative species interactions, as a function of different parameters: the average interaction strengths $\mu=\mu_{L}=\mu_{M}$, the connectivity $C_{M}$, $C_{L}$, the network size $S$, etc. In all these cases we found that, as long as the birth rates (given by Eq. (1) in the main text) remain positive during the evolution, extinctions are not observed (see Fig. S3 and S4).


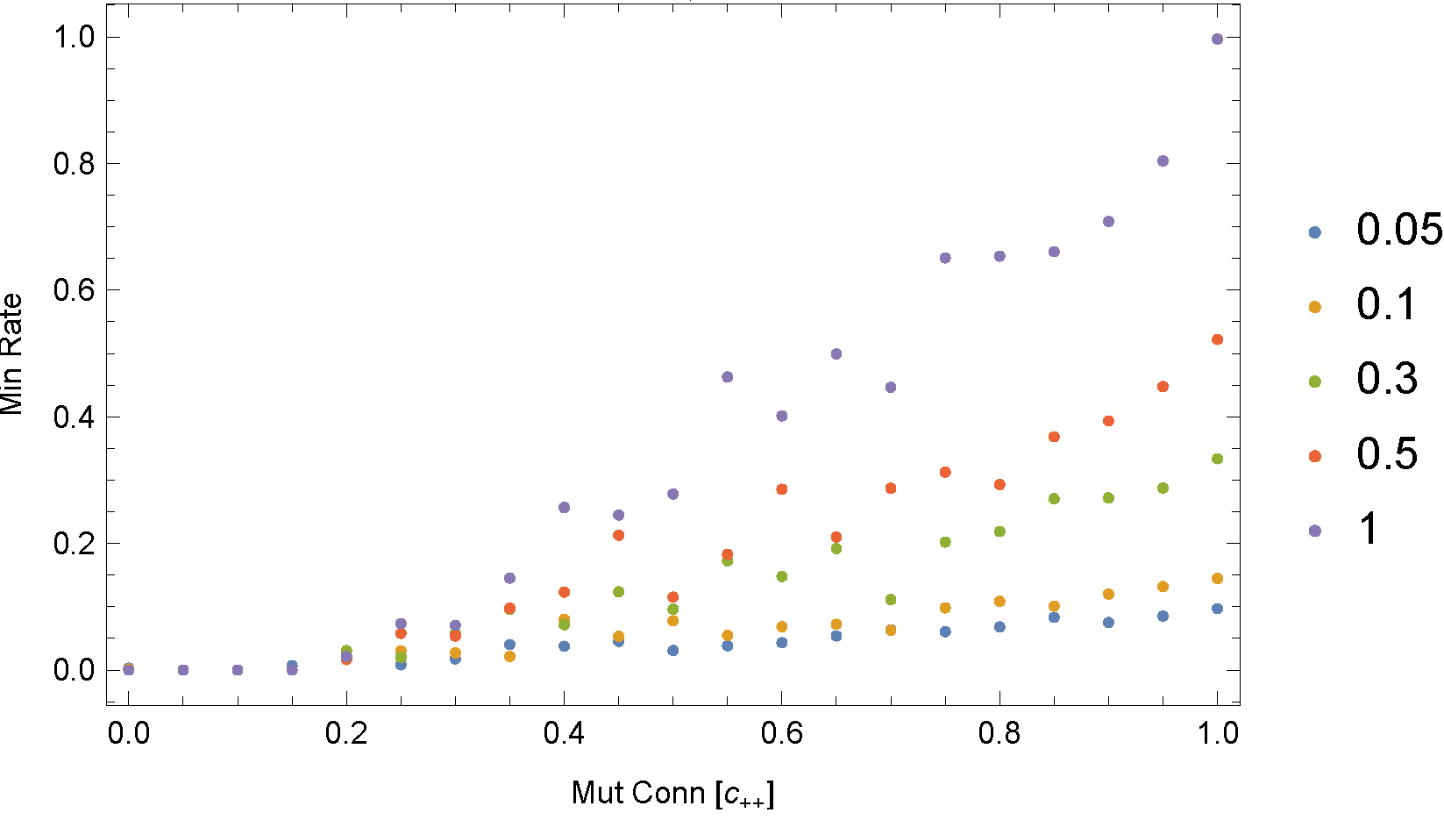


**Fig. S3. Min Rate defined as** $\min_{j=1,\ldots,S,t\geq0}\omega(j,\eta(t),M,L)$**.** The rates $\omega$ are given by Eq. (1) in the main text and $\eta(t)$ is its mean field solution of Eq. (2) in the main text, as a function of the connectivity of mutualistic $C_{M}=C_{++}$ and exploitative $C_{L}=1-C_{M}$ interactions for different average interaction strengths (colored points) $\mu=\mu_{M}=\mu_{L}=0.05,0.1,0.3,1$ and cooperative and exploitative intensities are . In all cases the distribution from which interaction strengths are drawn as explained in main text from a bivariate Gaussian distribution with mean $\mu$ and standard deviation $\sigma=0.01\mu$. The network size considered here is $S=20$. Similar results are found also for $S=50$ and $S=100$. The only cases where the birth rates (given by Eq. (1) in the main text) become negative during the mean field evolution, occur when exploitative interactions are dominant (region for $C_{M}<0.2$, $C_{L}>0.8$).


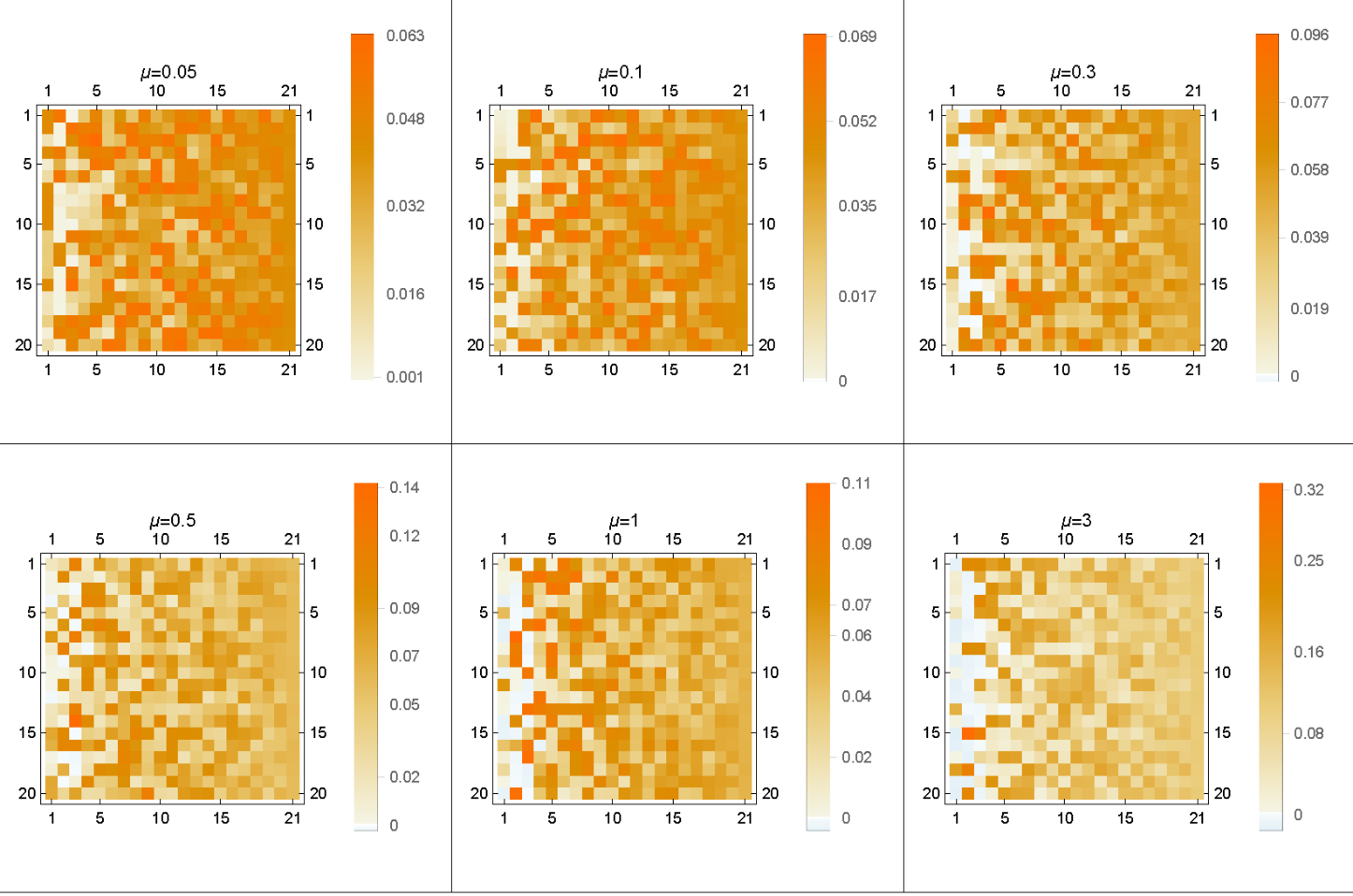


**Fig. S4. Populations of the species at the stationary state**. The dynamics is given by Eq. (2) in the main text and the parameters set are the same as Fig. S3. The $y$-axis denotes the species label (from 1 to 20), while the 21 points in the $x$-axis represents the 21 different connectivity configurations: from $C_{M}=0$ to $C_{M}=1$ with steps of $\Delta C_{M}=0.05$ and $C_{L}=1-C_{M}$. We numerically checked that as long as the birth rates (given by Eq. (1) in the main text) are positive, then no extinctions are observed (all species populations greater than zero).

**S5. Stability of the equilibria when**

As shown in the Materials and Methods of the main text, the exploitative interactions do not contribute to the stability of the fixed point in the large $S$ limit if . Here we present numerical simulations visualizing this result (see Fig. S5 and S6).


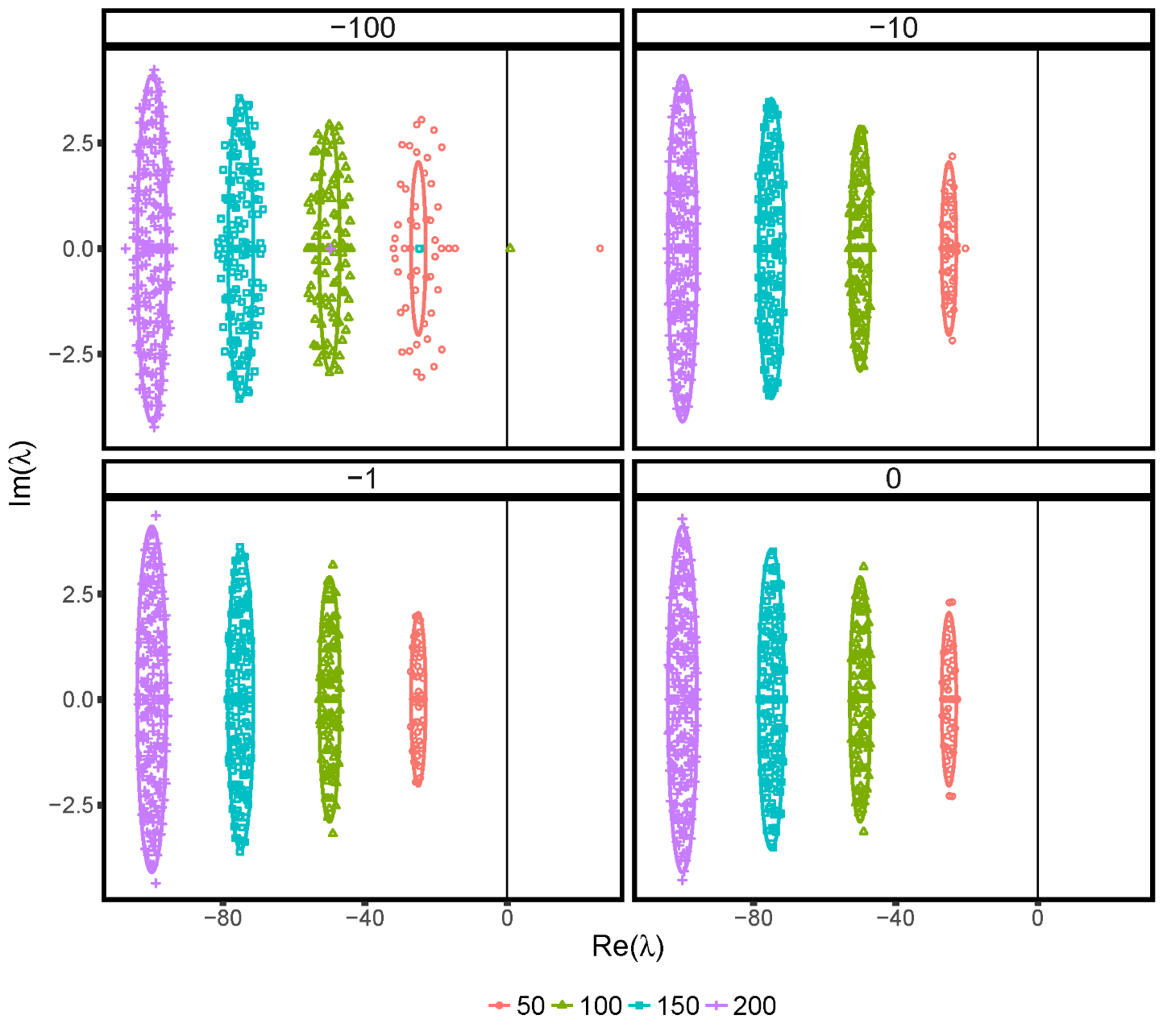


**Fig. S5. Spectrum of the Jacobian matrix for variable** **.** Different panels correspond to different values of (as denoted at the top of each inset), while and $C_{L}=C_{M}=0.5$, for all the simulations. The points are the eigenvalues of one Jacobian matrix obtained sampling at random the matrices $M$ and $L$, whose off-diagonal elements are both drawn uniformly between 0 and 1, while the lines indicate the analytical prediction obtained in the Materials and Methods of the main text, in the case . Colors and shapes correspond to different number of species ($S=50,100,150,200$ as denoted by the bottom legend). In all the cases, larger matrices turn out to be more stable. The black vertical line indicates the stability threshold.


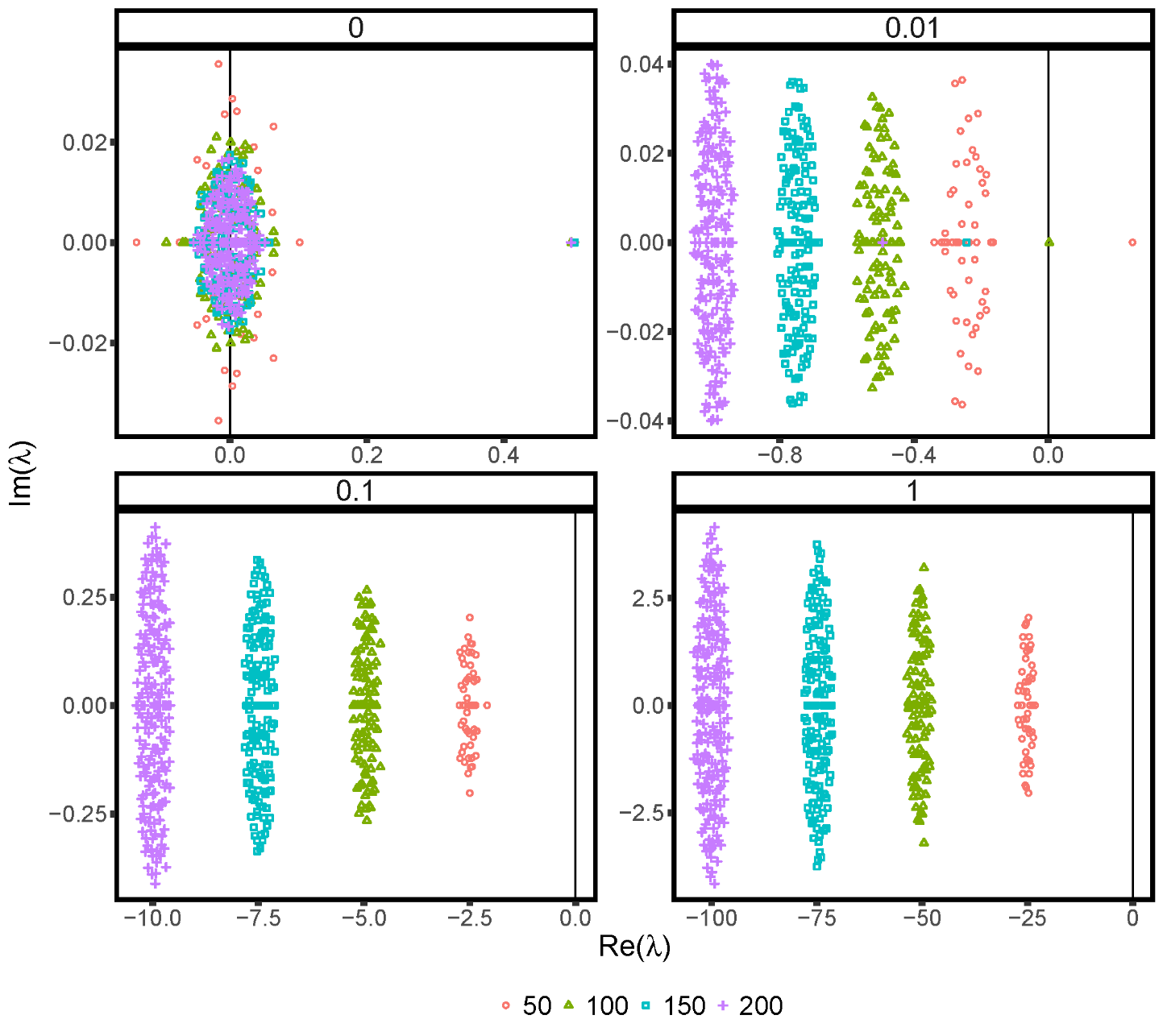


**Fig. S6. Spectrum of the Jacobian matrix for variable** **.** Same as in Fig. S5 but with and varying (as denoted at the top of each inset). Colors and shapes correspond to different number of species. When , the system is always unstable. As soon as a is considered, the spectrum shift on the left, making the system stable. It is important to observe that this happens even for very small values of . The minimum needed to stabilize the system is in fact expected to go to zero as the number of species $S$ increases ($S=50,100,150,200$ as denoted by the bottom legend). The off-diagonal elements of the matrices $M$ and $L$ are both drawn uniformly between 0 and 1.

**S6. Effect of** **Holling quadratic interactions**

We here provide the mathematical details showing that if in addition to mediated cooperation, we also add the classic direct mutualism through Holling quadratic interactions then we observe the violation of the complexity-stability paradox. Considering the quadratic term $\frac{G_{ij}x_{i}x_{j}}{1+gx_{i}x_{j}}$ (If $g=0$, it is Holling Type I; If $g>0$, it is Holling Type II), then the mean field equations of the system dynamics and its corresponding Jacobian matrix are:

Fig. S7 shows the fraction of extinctions obtained by simulating Eq. for different average interaction strengths as a function of the connectivity of the mediated cooperation ($c_{M}$). Here, we only consider feasible and stable solutions. We observe that as soon as $c_{M}$ is greater than 0.1, then basically all species coexist and stable. We find that as long as indirect mutualism is present, it will promote the ecosystem biodiversity and stability.


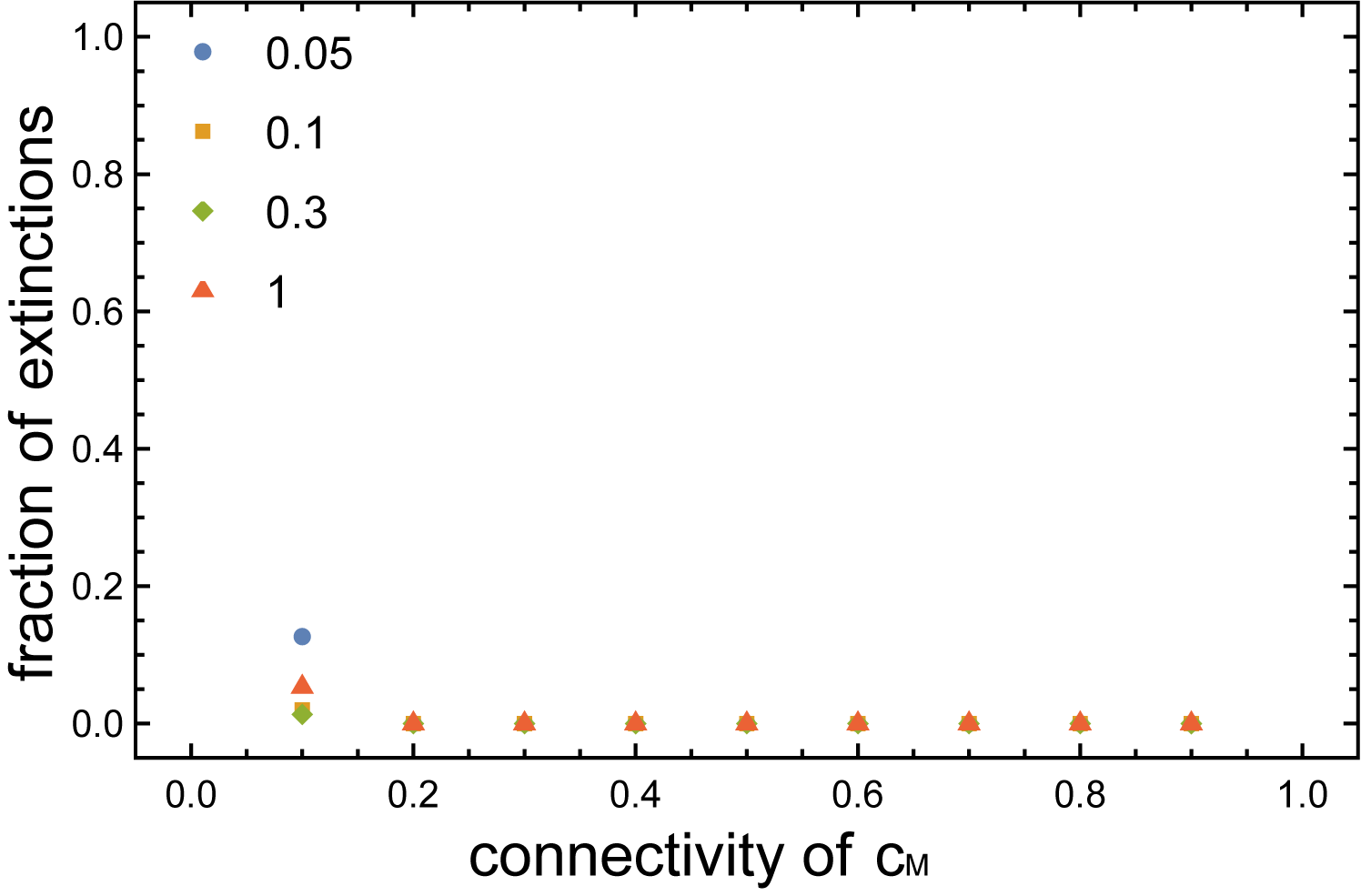


**Fig. S7.** **The fraction of extinctions for Holling quadratic interactions.** The fraction of extinctions when $c_{G}=1-c_{M}$ and $c_{M}$ varies from 0.1 to 0.9 as a function for different average interaction strengths $\mu_{M}=\mu_{G}$ (colored points). For simplicity, but without loss of generality, we here fix . Interaction strengths are drawn from a normal distribution with mean $\mu$ and standard deviation $\sigma=\mu/3$. The parameter of direct mutualistic term is set as $g=0.1$. The network size considered here is $S=50$ and each point is the mean of 8 realizations. Similar results are found also for $S=20,S=100$.

**S7. Mean field analysis for voter model with empty sites**

If we turn off exploitation (), the mean field equation without empty site () reads

where $s=1,\ldots,S$ represents different species, $\bar{\eta}^{s}$ is the average fraction of individuals of the $s$-th species, $M$ is the interaction matrix whose non-zero entries define the network of ecological interactions, $\theta$ is the Heaviside step function ($\theta(x)=1(0)$ for $x>0(x\leq0)$ ) and is the cooperation intensity (the average of the non-zero $M_{ij}$ is fixed to 1). For simplicity, we have omitted time dependence of $\bar{\eta}$. An intuitive derivation is as follows. The key point is that for $N$ large the evolution of the quantity $\bar{\eta}^{s}$ becomes deterministic because the noise is canceled in the macroscopic regime and in the thermodynamics limit the relative abundance converges to its mean. Then, observe that the dynamics of the relative abundance in the infinitesimal time $d t$ is simple as it can only decrease by $1/N$ when a site of kind $s$ change type or can increase by $1/N$ when the new symbol of a certain site is $s$.

We now extend the model presented in the main text introducing the possibility for a site to be empty. In our setting empty sites do not interact with species. Thus, the species rates remain unchanged after the introduction of empty sites. Thus, the species rates are the same as before whereas non-empty sites become empty with rate $\lambda$. In the case , the rate $\lambda$ has to be less than 1 otherwise empty sites will cover all the available space. The mean field equation becomes now:

Let us analyze the stationary mean-field equations for . In this case, the stable equilibrium for the empty sites is . Substituting in the equations for $\bar{\eta}^{s}$, we obtain

where $s=1,\ldots,S$. After the change of variable $\bar{\eta}'=(1-\lambda)\bar{\eta}$, the above Eq. reduces to the same equation as one would get for $\lambda=0$, i.e. in absence of empty sites the mean field equation becomes:

for $s=1,\ldots,S$, where $\sum_{j=1}^{S} \bar{\eta}^{j}(t)=1$ and is conserved by the dynamics.

In other words, when is small, the introduction of empty sites leads to stationary abundances which are trivially rescaled with respect to the case in absence of empty sites, as a consequence of the reduction of the available space.

**S8. Effect of rescaling of the species interaction matrix**

In the current model, each species has $S-1$ possible interactions which are nonzero with probability $c$. Thus, the average number of interactions a species has is $c(S-1)$ and these connections have the same average strength regardless of how many there are. Each cooperative connection in this model is interpreted physically as a cross-feeding relationship where species exchange nutrients. As the number of species increases, the species in this model also need to be producing ever larger pools of shared nutrients. In reality, if a species has a cross-feeding interaction with a larger number of species, the strength of the individual interactions decreases because the organism only secretes a finite amount of nutrient per unit biomass. Adding a constant effort hypothesis ^18^ is a reasonable option, although many works in the literature do not consider these types of tradeoffs ^19-21^. Nevertheless, we now show that all our results do not depend on the way we normalize the interactions strengths. For simplicity, but without loss of generality, to illustrate this point we here construct the interaction matrix as $A=M+L$ ($c_{M}+c_{L}=1$, i.e., we assume that each of the $S(S-1)/2$ links is either cooperative or exploitative, where $c_{M}>0,c_{L}\geq0$ are the connectivities of the matrix $M$, $L$ respectively. We normalize the strength of the interaction matrix $A$ for the degree, i.e., the new $A_{ij}^{norm}=A_{ij}/k_{j}^{out}$ where $k_{j}^{out}$ is the out-degree of the species $j$. We then compare the results of our model using both not-normalized and normalized version of $A$.

Fig. S8 shows that our results do not depend on the normalization of $A$. These results conform to our previous conclusions: If mutualism is present the fraction of extinctions is always very low for large enough $S$ and independent of the network weights.

**
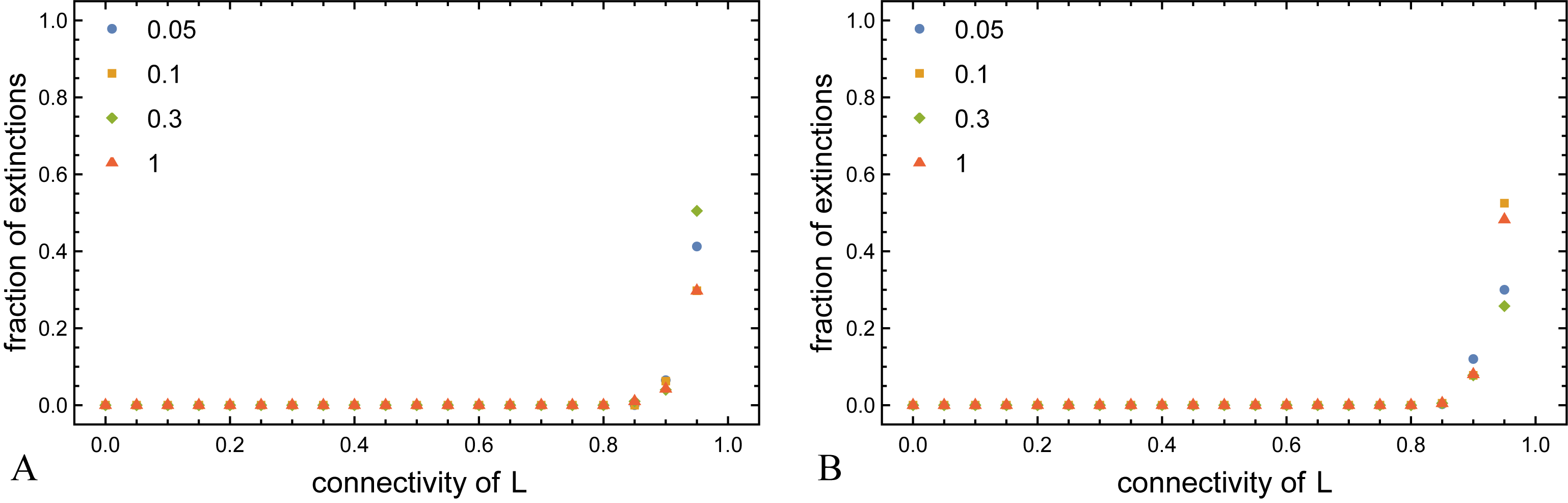
**

**Fig. S8. The fraction of extinctions for matrix with and without normalization. (A)** The fraction of extinctions for not normalized matrix $A$. **(B)** The fraction of extinctions for normalized matrix$A$ as a function of connectivity of matrix $L$ for different average interaction strengths (colored points) $\mu=\mu_{M}=\mu_{C}=0.05,0.1,0.3,1$ (see legend) and cooperative and exploitative intensities are . The distribution from which interaction strengths (before normalization) are drawn from a normal distribution with mean $\mu$ and standard deviation $\sigma=\mu/3$ The network size considered here is $S=50$ and each point is the mean of 8 realizations. Similar results are found also for $S=20,S=100$.

**Reference**

1 Morris, B. E. L., Henneberger, R., Huber, H. & Moissl-Eichinger, C. Microbial syntrophy: interaction for the common good. *FEMS microbiology reviews* **37**, 384-406 (2013).

2 Zelezniak, A. *et al.* Metabolic dependencies drive species co-occurrence in diverse microbial communities. *Proceedings of the National Academy of Sciences*, 201421834 (2015).

3 Tuncil, Y. E. *et al.* Reciprocal prioritization to dietary glycans by gut bacteria in a competitive environment promotes stable coexistence. *mBio* **8**, e01068-01017 (2017).

4 Goldford, J. E. *et al.* Emergent simplicity in microbial community assembly. *Science* **361**, 469-474 (2018).

5 Pacheco, A. R., Moel, M. & Segre, D. Costless metabolic secretions as drivers of interspecies interactions in microbial ecosystems. *bioRxiv*, 300046 (2018).

6 Monod, J. The growth of bacterial cultures. *Annual Reviews in Microbiology* **3**, 371-394 (1949).

7 Brückner, R. & Titgemeyer, F. Carbon catabolite repression in bacteria: choice of the carbon source and autoregulatory limitation of sugar utilization. *FEMS microbiology letters* **209**, 141-148 (2002).

8 Taillefumier, T., Posfai, A., Meir, Y. & Wingreen, N. S. Microbial consortia at steady supply. *eLife* **6**, e22644 (2017).

9 Posfai, A., Taillefumier, T. & Wingreen, N. S. Metabolic trade-offs promote diversity in a model ecosystem. *Physical review letters* **118**, 028103 (2017).

10 Seneta, E. *Non-negative matrices and Markov chains*. (Springer Science & Business Media, 2006).

11 Doering, C. R., Sargsyan, K. V. & Sander, L. M. Extinction times for birth-death processes: Exact results, continuum asymptotics, and the failure of the Fokker--Planck approximation. *Multiscale Modeling & Simulation* **3**, 283-299 (2005).

12 Faust, K. & Raes, J. Microbial interactions: from networks to models. *Nature Reviews Microbiology* **10**, 538 (2012).

13 Lima-Mendez, G. *et al.* Determinants of community structure in the global plankton interactome. *Science* **348**, 1262073 (2015).

14 Volkov, I., Banavar, J. R., Hubbell, S. P. & Maritan, A. Inferring species interactions in tropical forests. *Proceedings of the National Academy of Sciences* **106**, 13854-13859 (2009).

15 Ethier, S. N. & Kurtz, T. G. *Markov processes: characterization and convergence*. Vol. 282 (John Wiley & Sons, 2009).

16 Gardiner, C. W. *Handbook of stochastic methods for physics, chemistry and the natural sciences*. Vol. 25 (1986).

17 Penzl, T. Numerical solution of generalized Lyapunov equations. *Advances in Computational Mathematics* **8**, 33-48 (1998).

18 Suweis, S., Grilli, J. & Maritan, A. Disentangling the effect of hybrid interactions and of the constant effort hypothesis on ecological community stability. *Oikos* **123**, 525-532 (2014).

19 May, R. M. Will a large complex system be stable? *Nature* **238**, 413 (1972).

20 Allesina, S. & Tang, S. Stability criteria for complex ecosystems. *Nature* **483**, 205 (2012).

21 Suweis, S., Simini, F., Banavar, J. R. & Maritan, A. Emergence of structural and dynamical properties of ecological mutualistic networks. *Nature* **500**, 449 (2013).
